# Distinct physiological and functional responses in leaves and roots of co-occurring mangroves: contrasting invasive *Laguncularia racemosa* and native *Avicennia marina* under seasonal nutrient fluctuations

**DOI:** 10.3389/fpls.2026.1772780

**Published:** 2026-03-30

**Authors:** Wenna Wang, Xinxin Li, Haixiong Li, Yanjun Du

**Affiliations:** 1 School of Tropical Agriculture and Forestry (School of Agricultural and Rural Affairs, School of Rural Revitalization), Hainan University, Haikou, China; 2 Hainan Dongfang Black-faced Spoonbill Provincial Nature Reserve, Dongfang, China

**Keywords:** *Laguncularia racemosa*, Avicennia marina, mangroves, functional traits, invasive-native interactions, antioxidant enzymes, organ-specific responses, seasonal dynamics

## Abstract

**Introduction:**

Plant invasions threaten biodiversity and exacerbate climate impacts by disrupting ecosystems. Although stressors induce antioxidant and osmoregulatory defenses, field evidence from mangroves, key coastal systems undergoing rapid degradation, remains limited. In Hainan, China, the invasive *Laguncularia racemosa* displaces native *Avicennia marina*, but their organ-specific defense responses to seasonal fluctuations remain unclear.

**Methods:**

We measured antioxidant enzyme activities [superoxide dismutase (SOD), peroxidase (POD), and catalase (CAT)], osmolytes [proline (PRO) and soluble sugars (SS)], and malondialdehyde (MDA) levels in leaves, fine and coarse roots of *L. racemosa* and *A. marina* during Hainan’s dry and wet seasons, alongside leaf area, root conduit traits, and tissue nitrogen (N) concentrations.

**Results:**

While soil moisture and basic parameters remained stable, dry-season total soil N surged 4–7 fold. Leaves exhibited higher SOD and osmolyte levels than fine roots, while dry-season defenses diverged between a 9.5-fold leaf SOD increase in *L. racemosa* and enhanced root POD (2.6-fold) and CAT (1.6-fold in leaf, 2.2-fold in root) in *A. marina*. These enzyme and osmolyte patterns were consistent with seasonality rather than ontogeny. Notably, the upregulation of antioxidant defenses despite stable soil stressors, coupled with stable MDA levels and a general absence of negative enzyme–MDA correlations, suggests a plausible preventive defense model rather than reactive damage–repair. In the dry season, leaf area was generally constrained (except in mixed *L. racemosa*), and root conduit traits lacked consistent increases. Leaf and root N concentrations were largely stable (except in monospecific *A. marina*) and correlated more strongly with enzymes or osmolytes than with growth traits.

**Discussion:**

These findings probably suggest a priority allocation toward defense over growth. In mixed stands, dry-season edaphic facilitation enabled the invasive *L. racemosa* to pursue a high-risk hydraulic expansion, while the native *A. marina* maintained a conservative, safety-prioritized stability. Together, this contrasting behavior suggests that native community structure limits invasion by forcing invaders to undertake risky physiological trade-offs to sustain competitive dominance.

## 1 Introduction

Invasive plants are primary drivers of global biodiversity loss and ecosystem service degradation (Kuebbing and Nuñez, 2016; Beaury et al., 2023; Rilov et al., 2024). They suppress native species abundance and diversity, raise extinction risks, and restructure communities. Invasions also disrupt essential biogeochemical cycles, impose heavy economic costs, and impair human well-being (Egoh et al., 2020; Macêdo et al., 2024; Gallardo et al., 2024). Climate change exacerbates invasions through a pernicious feedback: projected warming and altered precipitation intensify invasion impacts, while invasion-driven degradation hastens climate feedbacks (Funk et al., 2014; Rilov et al., 2024). Invasive plants exhibited enhanced growth under water limitation and elevated CO_2_, with only slight variations in their photosynthetic and water-use traits (Sobuj et al., 2024). Yet most of the evidence derived from greenhouse or chamber studies (Anderson and Cipollini, 2013; Funk et al., 2016; Henn et al., 2019). Field stressors like drought and salinity create non-additive interactions (Cohen et al., 2021; Jiang et al., 2025), limiting predictions for woody invaders in multifaceted environments. Addressing this issue is therefore critical to sharpen invasion risk assessments, anticipate ecosystem responses to overlapping pressures, and inform precise management strategies (Vilà et al., 2024).

To unravel the invasion dynamics, it is essential to compare how native and invasive species modulate integrated antioxidant and osmoregulatory defenses under field conditions. Stressors such as drought, salinity, and chilling trigger excessive production of reactive oxygen species (ROS), causing oxidative damage to membranes, proteins, and nucleic acids (Boanares et al., 2020; Mittler et al., 2022; Fichman et al., 2023). In response, plants activate coordinated defense mechanisms: antioxidant enzymes, including superoxide dismutase (SOD), peroxidase (POD), and catalase (CAT), for ROS detoxification, along with osmoregulatory compounds such as proline (PRO) and soluble sugars (SS) for maintaining osmotic balance (Per et al., 2017; Boanares et al., 2020; Sun et al., 2020). Importantly, the distinct activation thresholds of these enzymes, where SOD and POD scavenge superoxide radicals (O_2_^-^) under mild stress while CAT mitigates severe H_2_O_2_ accumulation, provide a fine-tuned internal buffer that directly determines a plant’s physiological limits for enduring environmental fluctuations (Cruz de Carvalho, 2008; Boanares et al., 2020; Wang et al., 2022). At broader ecological scales, this capacity for internal biochemical buffering scales up to influence how species respond to seasonal environmental fluctuations, which are key drivers of ecological stability (Jiang et al., 2024). Long-term studies in grassland, forest and alpine ecosystems have demonstrated that seasonal variability in temperature, moisture and resource supply can erode community-level stability by weakening species asynchrony, amplifying temporal variation in productivity and slowing recovery from disturbance, thereby reducing ecosystem resilience (Bai et al., 2022; Jiang et al., 2024). Invasion ecology has emphasized that seasonal pulses of limiting resources, particularly nutrient fluctuations (*e.g*., nitrogen), act by transiently altering community invasibility through the creation of resource availability windows (Davis et al., 2000; Morecroft et al., 2016). Current perspectives on biotic resistance suggest that native community structure may influence invasibility not only through physical exclusion, but by exerting competitive pressures that force invasive species to alter their resource allocation strategies during these critical windows (Funk and Vitousek, 2007; Beaury et al., 2020). However, most work has inferred stability from whole-plant or community responses, leaving a critical knowledge gap: how the fine-scale partitioning of biochemical defenses provides the fundamental physiological basis for these macro-ecological patterns of resilience and invasion.

Crucially, evaluating stress-response strategies requires a whole-plant perspective. Because leaves face atmospheric drivers and light-induced oxidative risks, while roots navigate edaphic constraints and hydraulic demands, their defense priorities fundamentally differ (Chen et al., 2025). In the context of biological invasions, the capacity to optimally partition defensive investments between these functionally distinct organs determines a species’ ability to sustain aboveground carbon assimilation while simultaneously securing belowground resources under fluctuating conditions (Barros et al., 2021). Yet, the mechanisms underlying these tissue-specific biochemical coordinate patterns under seasonal stresses remain poorly understood, as previous research has relied instead on short-term, leaf-focused experiments (Sun et al., 2020). Furthermore, beyond biochemical defenses, an effective invasion strategy depends on how these metabolic patterns coordinate with functional traits. Specifically, because traits such as leaf area and root conduit dimensions govern the trade-offs between acquisition (*e.g*., carbon gain and hydraulic efficiency) and safety (*e.g*., transpirational control and hydraulic integrity) intrinsic to plant carbon and water dynamics (Choat et al., 2012; Anderegg et al., 2016), their functional integration with biochemical defenses is likely central to how plants navigate the metabolic demands of seasonal stress. Consequently, elucidating this coordination across the root-leaf continuum is essential to determine whether invasive species exploit seasonal pulses via high-risk acquisitive traits or via conservative structural adjustments that complement internal defenses, thereby uncovering the spatial resource allocation that drives invasion dominance over native species.

Mangrove ecosystems, with their high vulnerability to invasions, offer an ideal setting to examine these organ-specific physiological differences under field conditions. Covering ~13.4 million hectares worldwide, they offer irreplaceable services, such as biodiversity support, anchoring sediments, and sequestering carbon (C) at rates exceeding most terrestrial forests (Hochard et al., 2019; Bunting et al., 2022). Yet mangroves face escalating degradation globally, increasingly threatened by plant invasions (Goldberg et al., 2020; Bai et al., 2023). This threat intensifies in biodiversity hotspots like China’s Hainan Island, where seasonal dry-wet shifts amplify drought-salinity synergies and invaders inflict outsized disruptions on island biota relative to mainland ones (Pyšek et al., 2012). The invasive *Laguncularia racemosa*, introduced to Hainan in 1999, has spread rapidly due to fast growth and plasticity, which altering native mangrove communities (Bai et al., 2023). While previous greenhouse studies in China established the inherent stress tolerance and functional trait plasticity of *L. racemosa* (Gu et al., 2019; Lang et al., 2022; Zhang et al., 2023), whether these patterns persist under natural environmental variation (including fluctuating salinity, seasonal nutrient pulses, and competitive interactions) has not been systematically examined. Bridging the gap between idealized laboratory conditions and ecological realism, this field-based validation is essential to capture the non-additive interactions of multiple stressors that ultimately dictate the invasive success of *L. racemosa* in complex coastal environments. Furthermore, Hainan’s salinities exceed those in its Mexican native range by nearly threefold (Fazlioglu and Chen, 2020), contrasting with the co-occurring *Avicennia marina*, China’s dominant mangrove, which demonstrates superior halotolerance over *L. racemosa* (Nguyen et al., 2015; Méndez-Alonzo et al., 2016). These tolerance differences drive interspecific variations in water transport and regulation (Sobrado, 2000), indicating distinct antioxidant and osmoregulatory responses in *L. racemosa* and *A. marina* to Hainan’s seasonal stresses. Thus, the Hainan mangrove system provides a useful case study, showing how physiological differences between invaders and natives may help predict woody plant invasions in varying coastal ecosystems.

Building upon these distinct adaptive backgrounds, this study aimed to characterize the physiological and functional responses of invasive *L. racemosa* and native *A. marina* under seasonal nutrient fluctuations. Specifically, we investigated: (1) How do the antioxidant and osmoregulatory systems of *L. racemosa* and *A. marina* respond to seasonal environmental shifts, and what are the underlying biochemical mechanisms governing their seasonal oxidative damage dynamics under natural field conditions? (2) Do leaves and roots exhibit distinct spatial prioritization in their biochemical stress responses, and how are these organ-specific metabolic patterns coordinated with morphological constraints and root hydraulic architecture to mediate the fundamental trade-offs between resource acquisition and hydraulic safety? (3) Does *L. racemosa* and *A. marina* employ fundamentally divergent physiological and hydraulic strategies under prolonged natural stress, and how are the invader’s resource allocation patterns altered by the presence of native species during seasonal environmental fluctuations? To address this, we conducted a comparative field study sampling leaves, fine roots, and coarse roots of co-occurring individuals during Hainan’s dry and wet seasons, focusing on key biochemical stress markers (antioxidant enzymes, osmolytes, and MDA). Crucially, to distinguish these seasonal factor responses from potential intrinsic growth-related changes, we simultaneously monitored morphological, anatomical, and biogeochemical traits, specifically leaf area, root conduit traits, and nitrogen (N) concentration in both leaves and roots, as reference indicators of intrinsic growth trends. By answering these questions, we aim to elucidate the comparative ecophysiological mechanisms that underpin the distinct stress-response strategies of these invasive and native woody species in dynamic coastal environments.

## 2 Materials and methods

### 2.1 Study site and experimental setup

The study was conducted at the Sibi Bay Mangrove Reserve (108°37′–108°40′ E, 19°11′–19°13′ N) in Hainan, China. This tropical monsoon maritime region supports monodominant stands of *A. marina* and invasive *L. racemosa*. The site is characterized by a mean annual temperature of 24.6 °C (January 18.4 °C, July 29 °C) and mean annual precipitation of 1018.3 mm. Tides are irregular semidiurnal, reaching mean high-water levels of 1.5 m in the wet season and 1.0 m in the dry season.

Experimental stands were established on coastal saline soils (0.5–3 m a.s.l.) using one-year-old seedlings planted at a density of 1 m × 1 m (~10,000 seedlings/ha). A completely randomized design was employed with three stand types: *A. marina* monoculture, *L. racemosa* monoculture, and a mixed stand of both species (1:1 mixture). Replicate 10 × 10 m plots were established for each stand type, separated by buffer zones of >20 m. Failed seedlings were replaced immediately after planting to ensure consistent competition intensity. Sampling was conducted during the wet season (August 2023, >210 mm monthly rainfall) and dry season (February 2024, <15 mm monthly rainfall) to capture contrasting environmental drivers. Basic soil properties for the 0–20 cm layer are summarized in **Table 1**. To evaluate the functional impacts of seasonal shifts, this study focused on organ-level physiological responses as indicators of stress exposure. While soil and tissue total N were monitored as background indicators, a comprehensive nutrient profiling (*e.g*., P and K) was not conducted. Consequently, seasonal patterns are interpreted as correlational rather than mechanistically attributing responses to specific nutrient or moisture drivers.

**Table 1:** Soil physicochemical properties of the topsoil (0–20 cm) in monospecific and mixed stands of *Laguncularia racemosa* and *Avicennia marina* during the dry and wet seasons.

Season	Stand type	Total nitrogen (g/kg)	Total carbon (g/kg)	C:N ratio	Electric conductivity /(mS·cm-1)	pH	Gravimetric Water content/%
Wet season	LR-P	0.05 ± 0.01 X**	4.25 ± 0.85 X	84.93 ± 5.04 X**	5.75 ± 0.93 X	7.43 ± 0.08 X	13.56 ± 1.04 X
	AM-P	0.06 ± 0.01 X**	3.61 ± 0.78 X	55.67 ± 3.79 Y**	3.63 ± 0.55 X	7.29 ± 0.06 X	14.48 ± 1.89 X
	LR-AM-M	0.07 ± 0.01 X**	4.15 ± 0.46 X	59.38 ± 2.71 Y**	3.59 ± 0.37 X	7.25 ± 0.06 X	15.49 ± 1.19 X
Dry season	LR-P	0.27 ± 0.03 C**	4.83 ± 0.52 A	19.81 ± 1.35 A**	5.75 ± 1.63 A	7.34 ± 0.12 A	16.51 ± 2.03 A
	AM-P	0.47 ± 0.03 A**	4.70 ± 0.59 A	9.84 ± 0.93 B**	4.16 ± 0.76 AB	7.27 ± 0.09 A	16.21 ± 2.74 A
	LR-AM-M	0.36 ± 0.01 B**	3.29 ± 0.21 A	9.32 ± 0.66 B**	3.47 ± 0.5 B	7.22 ± 0.05 A	16.01 ± 0.83 A

LR-P, *Laguncularia racemosa monospecific stand*; AM-P, Avicennia marina monospecific stand; LR-AM-M, *L. racemosa-A. marina mixed stand*. Different uppercase letters denote significant differences among stand types within the same season, X, Y for wet season and A, B, C for dry season, while single (*) and double (**) asterisks indicate significant seasonal differences within each stand type at the *P* < 0.05 and *P* < 0.01 levels, respectively, based on a linear mixed model. Some of the soil physicochemical data presented here were derived from previously published measurements for the 0–10 cm and 10–20 cm soil layers (Yang et al., 2026). In the present study, these data are reported as the mean values for the 0–20 cm topsoil to match the analytical framework of this manuscript and to enable an integrated comparison among stand types and seasons.

### 2.2 Field sampling

In both the wet and dry seasons, three healthy, uniform saplings (approx. 4–5 years old) per species were randomly selected from three replicate plots per stand type. In mixed stands, individuals of both species were sampled. To minimize diurnal variation, all sampling was conducted between 9:00 and 11:00 a.m. under consistent weather conditions.

From each selected tree, fully expanded, sun-exposed leaves were collected from three different canopy orientations. To obtain a single composite sample representative of the experimental unit (plot), leaf samples from the three individual trees were pooled into a single composite sample per plot for physiological assays. A subset of leaves was separated for leaf area measurement, while concurrent sampling for total N synchronized nutrient status with morphological and biochemical assessments.

At the same time, roots were excavated from the upper 0–20 cm soil layer, corresponding to the primary oxic rooting zone (Aritsara et al., 2022). Specifically, primary anchoring roots (>2 cm diameter) were carefully exposed, and intact root branches containing both fine (0–2 mm) and coarse (2–5 mm) orders were harvested. From the same selected trees, three distinct sets of root samples were prepared: (1) Anatomical samples: multiple root segments per plant were gently rinsed in seawater and immediately pooled into a single bottle containing formalin-aceto-alcohol (FAA, 90 ml of 50% ethanol, 5 ml of 100% glacial acetic acid, and 5 ml of 37% methanol); (2) Physiological samples: additional root tissues were harvested, rinsed, and pooled across the three trees to form composite samples in Ziploc^®^ bags; and (3) Hydration samples: a parallel set of root branches was harvested for tissue water content (TWC) analysis; (4) Nutrient samples: fine and coarse root classes were mixed and pooled into a single composite sample per plot to represent the integrated N status of the root system.

Rhizosphere soil was sampled concurrently from three randomly selected points within each plot to capture spatial heterogeneity. After removing surface litter, soil cores were collected from two depth intervals (0–10 cm and 10–20 cm). To prevent cross-contamination and allow for potential intra-plot variability assessment, samples from each point and depth were packed individually into Ziploc^®^ bags (i.e., 3 points × 2 depths per plot). All fresh plant and soil samples were transported on dry ice to the laboratory within 2 h.

### 2.3 Quantification of plant and soil traits

Root samples preserved in FAA solution (for anatomy) and Ziploc^®^ bags (for physiology) were separated into fine (<2 mm, absorptive) and coarse (2–5 mm, transport) functional classes using a digital caliper (0.01 mm accuracy), based on established morphological criteria (Pregitzer et al., 2002; Yuan et al., 2011; Roumet et al., 2016).

For root anatomy, approximately 30 individual roots per diameter class were analyzed for every season, species, and stand type. Samples were paraffin-embedded, sectioned transversely at 8 μm, and stained with safranin-fast green (Wang et al., 2018). Using an Olympus BX-51 microscope and Motic Images Advanced 3.2 software, we quantified root diameter and stele conduit characteristics (diameter and count). These individual measurements were subsequently aggregated to calculate plot-level means (see Section 2.4).

Enzymatic and metabolite assays were conducted on homogeneous root and leaf tissue samples. Enzyme activities (SOD, POD, CAT) were determined from 0.1g fresh tissue homogenized in 50 mM phosphate buffer (pH 7.8) and centrifuged (8000 g, 10 min, 4 °C). Assays were performed using commercial kits (Nanjing MolFarming, China) on a SpectraMax 190 reader: SOD via nitroblue tetrazolium inhibition (560 nm, 37 °C), POD via guaiacol oxidation (470 nm), and CAT via H_2_O_2_ decomposition (405 nm, 37 °C). Activities were expressed as U g^-^¹ fresh weight (FW). To evaluate potential dehydration bias in FW-normalized assays, TWC was determined using parallel samples (*n* = 2 composites per class). Fresh tissues were weighed immediately to record FW, and then oven-dried (70 °C) to a constant mass to determine dry weight (DW), with TWC calculated as [(FW-DW)/FW]×100%. Conversely, metabolites (MDA, PRO, SS) were quantified using oven-dried (70 °C) samples extracted in 80% ethanol, following standard protocols (TBA method for MDA, acidic ninhydrin for PRO, anthrone-sulfuric acid for SS). Assays followed standard protocols with three biological replicates.

Soil physicochemical properties were measured to characterize the rooting environment. Soil pH and electric conductivity were measured in a 1:2.5 suspension using a multi-parameter analyzer (JC-TR-4G, China), while soil water content was determined gravimetrically after drying at 105 °C. Total C and total N concentrations for both soil and plant tissues (dried at 65 °C, ground, and 100-mesh sieved) were quantified using an elemental analyzer (Vario EL Cube, Elementar, Germany) to calculate C:N ratios.

### 2.4 Data analysis

To rigorously align the statistical analysis with the experimental design, where the “plot” represents the fundamental unit of biological independence, we aggregated all sub-sample measurements to the plot level. Given that individual root segments and soil sampling points within a single plot are spatially correlated, treating them as independent replicates would inflate the error degrees of freedom. Consequently, we calculated the arithmetic mean of conduit traits for each plot (*n* = 3). Similarly, for soil properties, measurements from the three sampling points within each plot were averaged to yield a single representative value per depth per plot. Subsequently, preliminary LMM analyses revealed no significant effect of depth on key edaphic factors (e.g., electric conductivity and soil water content, *P* > 0.05). Therefore, we calculated the weighted mean of the 0–10 cm and 10–20 cm layers to represent the integrated conditions of the primary rooting zone (0–20 cm). This aggregation step ensured that the resolution of anatomical and environmental datasets was commensurate with the physiological matrices, providing a robust framework for subsequent mixed-effects modeling. Accordingly, some soil physicochemical data associated with our previous study (Yang et al., 2026) were restructured here as integrated values for the 0–20 cm layer to support the present analysis.

To evaluate both species-specific plant responses and soil properties while accounting for the hierarchical structure and temporal correlation of the data, LMMs were fitted using the lme4 package in *R*. To address our primary objective, we constructed a core global model to assess physiological variation, specifically antioxidants (SOD, POD, CAT), osmoregulators (SS, PRO), and MDA. This model designated “forest type” (four levels: *L. racemosa* monoculture, *L. racemosa* mixed, *A. marina* monoculture, *A. marina* mixed), “season”, and “organ” as fixed effects. This formulation inherently accounts for both species identity and mixing status within a single factor, allowing for simultaneous assessment of interspecific differences (e.g., *L. racemosa* mono vs. *A. marina* mono) and intraspecific mixing effects (e.g., *L. racemosa* mono vs. *L. racemosa* mixed). Furthermore, to rigorously isolate seasonal responses from potential ontogenetic confounding, supplementary LMMs formally tested the main and interactive effects of season, tree species, and stand type (monospecific vs. mixed) on these physiological markers alongside comprehensive leaf and root morphological, anatomical, and biochemical traits. In all models, “Plot ID” was included as a random intercept. Assumptions of normality and homoscedasticity were verified via residual plots; data were log-transformed where necessary. Pairwise comparisons for significant fixed effects were conducted using estimated marginal means (emmeans package), with *P* values adjusted using the Tukey method.

Although concurrent stable soil moisture indicated overall hydration stability, the limited initial sample size (*n* = 2 composite samples) for root tissue water content could not definitively preclude subtle tissue water fluctuations, suggesting that the unadjusted FW-based results should be interpreted with caution if evaluated in isolation. To maximize statistical transparency, descriptive statistics (mean, SD, and CV%) were calculated. Given that a sample size of two per season is insufficient for meaningful confidence interval estimation, seasonal data were pooled (*n* = 4 per species-organ-stand combination) to construct 95% confidence intervals (CIs). This pooling approach, while maximizing the statistical utility of the dataset, is predicated on the assumption of seasonal stability, which is precisely what the analysis aims to evaluate. This creates a circular logic: stability justifies pooling, and the pooled intervals are then presented as evidence of stability. However, because no robust statistical descriptor can be derived from an *n* of two, these pooled CIs are presented not as proof of stability, but as the most robust descriptor of central tendency and variance achievable given the unavoidable constraints of the dataset. Beyond sampling constraints, to validate the primary FW enzymatic measurements against potential seasonal tissue hydration biases, a mathematical sensitivity analysis was performed. Following established protocols (Kováik, 2021), season- and group-specific dry-to-fresh mass conversion factors, derived from their respective mean water contents, were applied to recalculate the original FW data into DW equivalents, providing a reliable diagnostic reference to verify the consistency of seasonal physiological trends when physical re-measurement was constrained.

Principal component analysis (PCA) was used to associate morphological and anatomical traits, specifically leaf area in leaves, root mean conduit diameter (CD) and conduit number (CN), and N concentration in both leaves and roots, with physiological variables (*i.e*., antioxidant enzymes, osmolytes, and MDA). To visualize distinct functional patterns across organs, separate PCAs were performed for leaf, fine root, and coarse root tissues within each species. Each PCA was based on plot-level data with a sample size of *n* = 6 independent replicates per species and organ. These replicates pooled data from both monospecific and mixed stands in order to capture the full range of trait variation driven by planting pattern. To allow direct comparison with root N concentration in the PCA, values for all other root physiological and anatomical variables were calculated as weighted means of coarse−root and fine−root measurements, ensuring all traits were analyzed at a consistent whole−root organ level. Additionally, Pearson’s correlation coefficients (*r*) were calculated to quantify the internal relationships among physiological variables within each species, organ, and season. All analyses were performed in the R environment (v.4.2.0), and figures were generated using Origin 2021.

## 3 Results

### 3.1 Seasonal variation in physiological, morphological and anatomical traits

While most soil properties remained seasonally stable, N-related soil stoichiometry exhibited pronounced seasonal dynamics. Basic parameters (water content, electrical conductivity, pH, and total C) remained seasonally stable (*P* > 0.05, **Table 1**). Conversely, nutrient stoichiometry was highly dynamic. Specifically, dry-season total N surged to levels 5–8 times higher than in the wet season (*P* < 0.01), causing a marked reduction in the C:N ratio.

**Figure 1:**
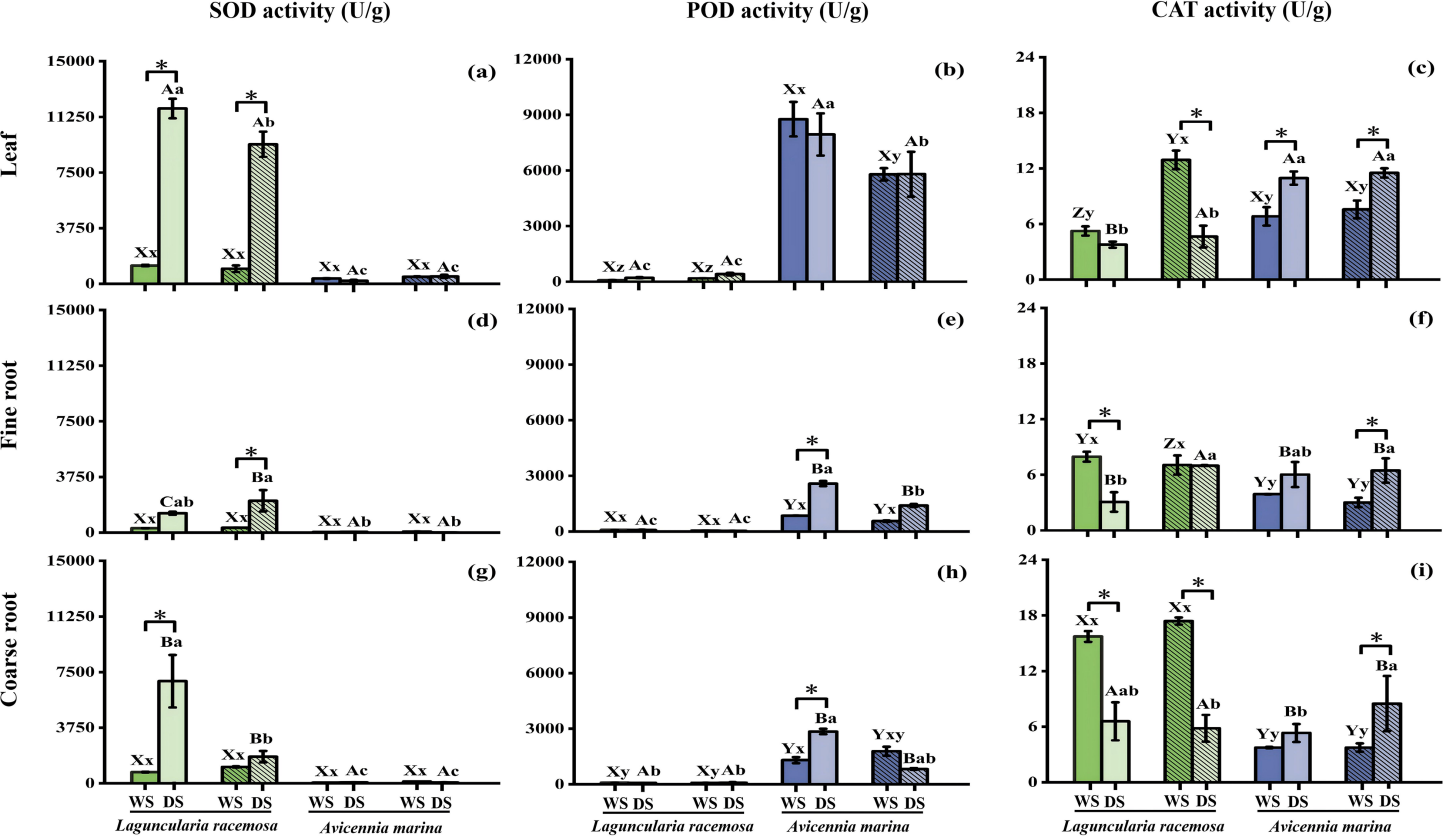
Activities of superoxide dismutase (SOD), peroxidase (POD), and catalase (CAT) in leaves **(a-c)**, fine roots **(d-f)**, and coarse roots **(g-i)** of *Laguncularia racemosa* (green) and *Avicennia marina* (purple) in monospecific and mixed stands during wet (WS) and dry (DS) seasons (*n* = 3). Solid-filled bars represent monospecific stands, while hatched bars denote mixed stands. Error bars represent standard errors. Different lowercase letters denote significant differences among the four stand–species combinations within the same organ and season, x, y, z for wet season, a, b, c, d for dry season; uppercase letters denote differences among organs within the same stand–species combination and season, X, Y, Z for wet season, A, B, C for dry season. Asterisks (*) indicate seasonal differences within the same stand–species combination and organ (*P* < 0.05).

**Figure 2:**
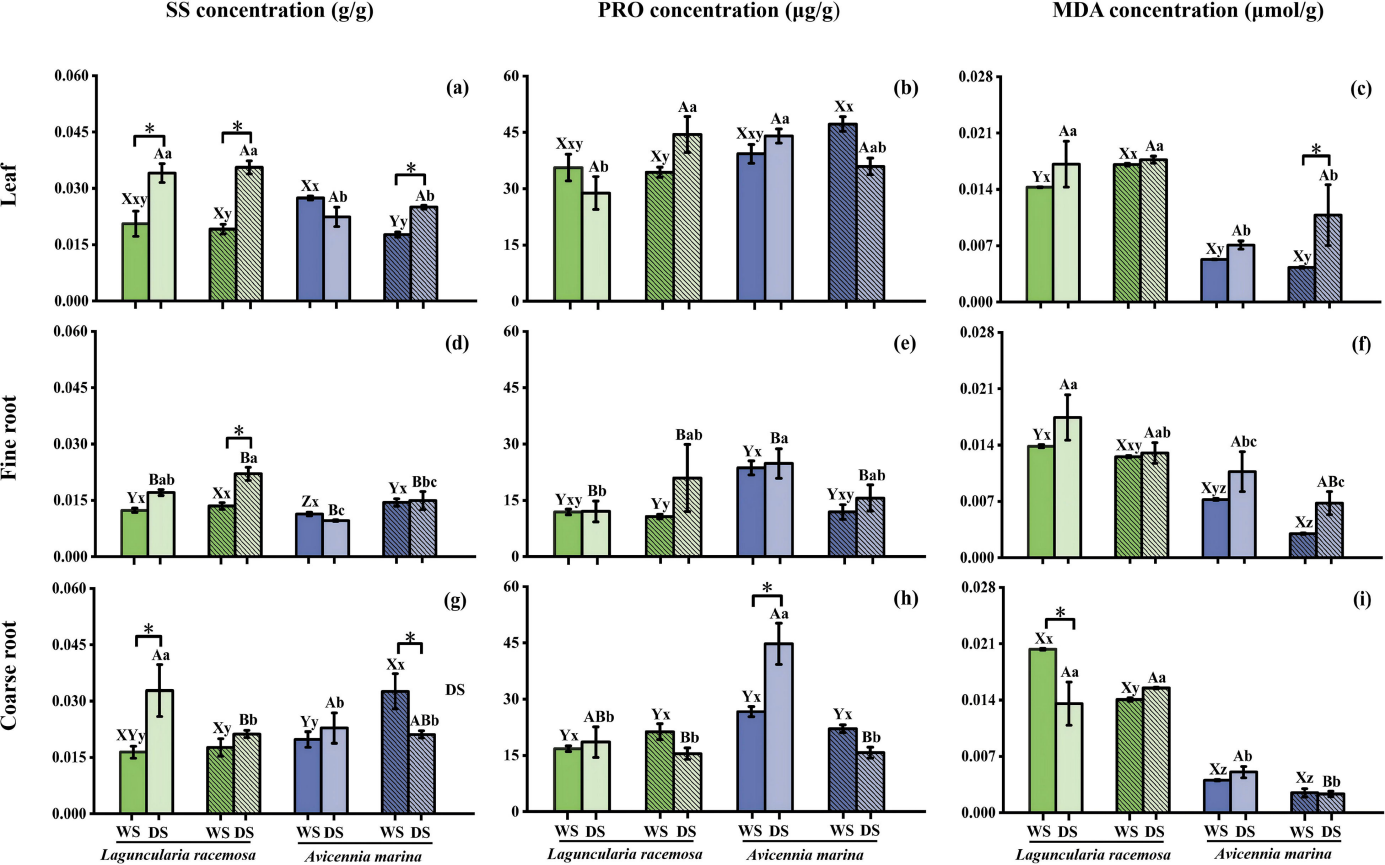
Soluble sugar (SS), proline (PRO), and malondialdehyde (MDA) concentrations in leaves **(a-c)**, fine roots **(d-f)**, and coarse roots **(g-i)** in monospecific and mixed stands of *Laguncularia racemosa* and Avicennia marina during the wet (WS) and dry (DS) seasons (*n* = 3). Asterisks (*) indicate seasonal differences within the same stand–species combination and organ (P < 0.05). Refer to **Figure 1** for bar color and hatching definitions, as well as statistical significance notations (lowercase letters and uppercase letters).

Seasonal variations in SOD activity and SS concentration were significant (*P* < 0.01) and were largely driven by *L. racemosa* (**Table 2**; **Figure 1**, **2**). Specifically, the dry season significantly elevated SOD activity and SS concentration in *L. racemosa* leaves (both stands), fine roots of mixed stands, and coarse roots of monospecific stands (**Figures 1a, d, g**, **2a, d, g**). While the dry season reduced CAT activity in coarse root of both stands, leaves of mixed stands, and fine roots of monospecific stands (**Figures 1c, f, i**). In contrast, SOD activity in *A. marina* remained seasonally stable in leaves and roots of monospecific stands (**Figures 1a, d, g**). Instead, seasonal responses in *A. marina* were characterized by increased CAT activity during the dry season in leaves (both stands), fine and coarse roots of mixed stands, as well as elevated root POD activity in monospecific stands (*P* < 0.05, **Figures 1e, h, c, f, i**). However, POD activity in *L. racemosa*, along with PRO and MDA concentrations in both species, showed no significant seasonal variation (**Table 2**, **Figures 1**, **2**).

**Table 2:** Linear mixed model results for the effects of season, organ and forest type on SOD, POD, CAT activities and SS, PRO, MDA concentrations in *Laguncularia racemosa* and *Avicennia marina*.

Source of Variation		SOD activity		POD activity		CAT activity		SS concentration		PRO concentration		MDA concentration	
	*df*	*F* value	*P* value	*F* value	*P* value	*F* value	*P* value	*F* value	*P* value	*F* value	*P* value	*F* value	*P* value
Season	1	159.42	***P* < 0.01**	5.50	**0.02**	8.93	***P* < 0.01**	21.33	***P* < 0.01**	0.98	0.33	4.01	**0.05**
Organs	2	66.79	***P* < 0.01**	138.13	***P* < 0.01**	16.32	***P* < 0.01**	41.56	***P* < 0.01**	64.10	***P* < 0.01**	1.97	0.15
Forest Type	3	91.53	***P* < 0.01**	131.44	***P* < 0.01**	7.58	***P* < 0.01**	2.25	0.09	8.81	***P* < 0.01**	47.33	***P* < 0.01**
Season×Organs	2	41.48	***P* < 0.01**	2.33	0.11	7.37	***P* < 0.01**	2.71	0.08	0.66	0.52	2.84	0.07
Season×Forest Type	3	61.12	***P* < 0.01**	1.43	0.25	39.16	***P* < 0.01**	11.23	***P* < 0.01**	3.06	**0.04**	0.87	0.46
Organs×Forest Type	6	19.74	***P* < 0.01**	43.66	***P* < 0.01**	12.57	***P* < 0.01**	3.60	***P* < 0.01**	1.85	0.11	2.31	**0.05**
Season×Organs×Forest Type	6	18.03	***P* < 0.01**	1.67	0.15	5.36	***P* < 0.01**	3.92	***P* < 0.01**	2.25	0.06	1.13	0.36

The forest types encompassed four levels, consisting of monospecific and mixed stands of both *L. racemosa* and *A. marina*. Abbreviations defined in **Figures 1** and **2**. Bold font indicates significance at P < 0.05.

Importantly, overall root tissue water content remained broadly stable across species, root classes, or seasons (**Table 3**). Even in specific root cohorts exhibiting higher variance (CV ≤ 23.3%), dry-season mean hydration remained comparable to or higher than wet-season levels. A mathematical sensitivity analysis cross-validating FW to DW activities showed that DW normalization preserved all of the significant seasonal differences observed in the primary FW data (**Supplementary Figure S1**). Additionally, this analysis revealed three additional significant seasonal differences compared to initial FW analyses: POD in both fine and coarse roots of the mixed *A. marina* stand, and CAT in fine roots of the mixed *L. racemosa* stand.

**Table 3:** Seasonal variation in tissue water content, including means, SD, CV%, and 95% confidence intervals (CIs), of fine (<2 mm) and coarse (2–5 mm) roots in *Laguncularia racemosa* and *Avicennia marina* across different forest types.

Forest type	Organ	Season	Means (%)	SD	CV (%)	95% CIs
Avicennia marina						
Monospecific stand	Fine Root	Wet	69.9	3.2	4.6	[56.3, 89.7]
		Dry	76.0	16.5	21.7	
	Coarse Root	Wet	80.6	1.0	1.3	[69.1, 83.9]
		Dry	72.8	0.9	1.3	
Mixed stand	Fine Root	Wet	74.2	14.3	19.2	[62.9, 92.6]
		Dry	81.6	2.3	2.8	
	Coarse Root	Wet	74.2	3.0	4.0	[58.0, 88.5]
		Dry	72.5	16.9	23.3	
Laguncularia racemosa						
Monospecific stand	Fine Root	Wet	78.9	14.5	18.4	[66.2, 92.3]
		Dry	79.3	0.3	0.3	
	Coarse Root	Wet	79.0	0.5	0.6	[76.5, 85.5]
		Dry	82.9	2.6	3.1	
Mixed stand	Fine Root	Wet	72.9	2.1	2.9	[66.2, 89.8]
		Dry	83.8	6.1	7.3	
	Coarse Root	Wet	75.1	3.2	4.3	[70.3, 79.7]
		Dry	74.8	4.5	6.1	

Root samples for tissue water content determination were collected concurrently with those for physiological measurements. Seasonal metrics (means, SD, and CV%) are based on two composite samples per species-organ-stand combination (n=2) and should be interpreted with caution due to the limited sample size. To enhance statistical power, cross-seasonal data were pooled (n = 4) to calculate 95% CIs.

**Figure 3:**
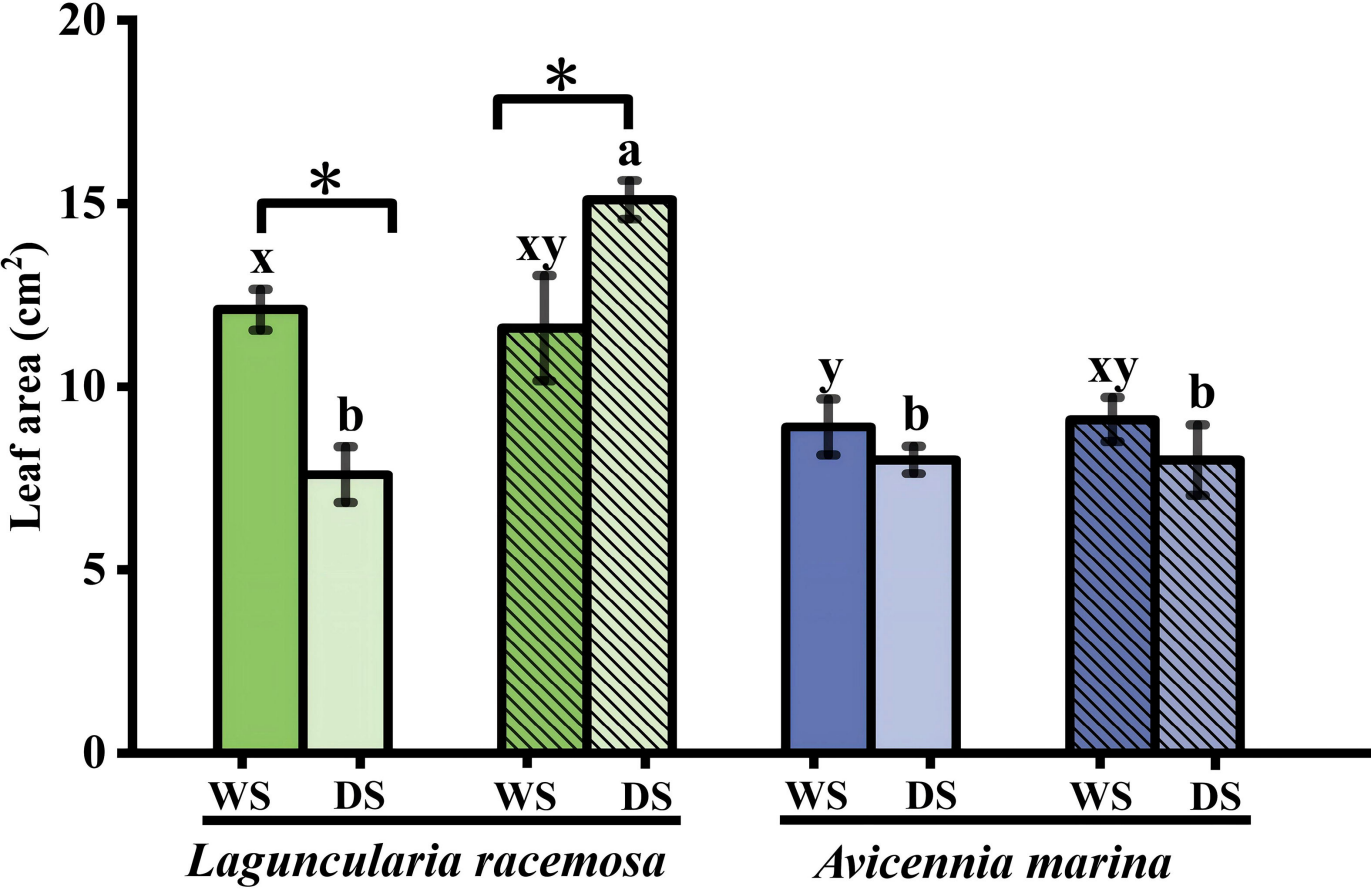
Leaf area of *Laguncularia racemosa* and *Avicennia marina* in monospecific and mixed stands during wet (WS) and dry (DS) seasons (*n* = 3). Asterisks (*) indicate seasonal differences within the same stand–species combination and organ (P < 0.05). Refer to **Figure 1** for bar color and hatching definitions, as well as lowercase letters indicating statistical significance.

**Figure 4:**
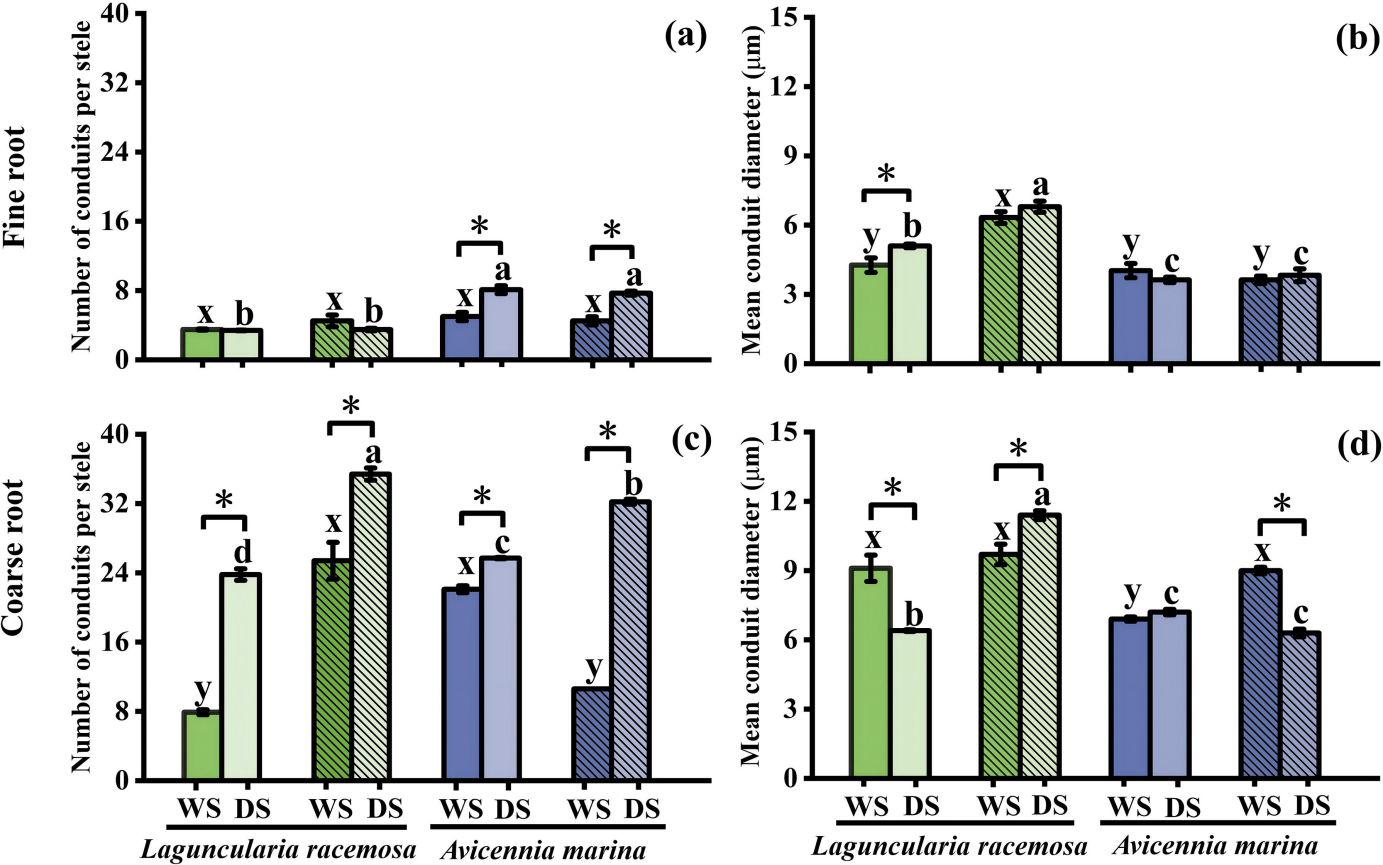
The number of conduits per stele and mean conduit diameter in fine roots **(a, b)** and coarse roots **(c, d)** in monospecific and mixed stands of Laguncularia racemosa and *Avicennia marina* during the wet (WS) and dry (DS) seasons (*n* = 3). Asterisks (*) indicate seasonal differences within the same stand–species combination and organ (*P* < 0.05). Refer to **Figure 1** for bar color and hatching definitions, as well as lowercase letters indicating statistical significance.

Leaf area in monospecific *L. racemosa* was significantly higher in the rainy season, whereas in mixed stands it was higher in the dry season (*P* < 0.05), while *A. marina* showed no significant variation (*P* > 0.05, **Figure 3**). Fine root conduit traits generally remained seasonally stable, with exceptions limited to increased conduit diameter in monospecific *L. racemosa* and number in *A. marina* (**Figures 4a, b**). Conversely, coarse roots showed stronger seasonal responses: conduit number increased universally across all species in the dry season (**Figure 4c**). By contrast, coarse root diameter generally remained stable or decreased, except for an increase in mixed *L. racemosa* (**Figure 4d**). *L. racemosa* maintained consistent tissue N concentrations across seasons regardless of planting patterns (*P* > 0.05), whereas *A. marina* showed significant leaf N enrichment only in monospecific stands during the dry season (*P* < 0.05, **Supplementary Table 1**). To provide stronger statistical support for distinguishing seasonality from ontogeny, a supplementary unified LMM was implemented (season × species × stand type, **Supplementary Tables 2**, **3**). Within this framework, seasonal main effects were significant for SOD, MDA, and SS (*P* < 0.05), but not for POD, CAT, or PRO (*P* > 0.05). Among morphological and anatomical traits, seasonal effects were non-significant for leaf area and fine-root conduit diameter, but were significant for fine-root conduit number, coarse-root conduit number and diameter, and root N concentration (**Supplementary Table 2**, **3**).

### 3.2 Organ‐specific differences in physiological, morphological and anatomical traits

In both monospecific and mixed stands, leaf SOD activity in *L. racemosa* (dry season) and POD activity in *A. marina* (both seasons) significantly exceeded fine and coarse root levels (*P* < 0.05, **Figures 1a, b, d, e, g, h**). Similarly, leaf PRO and SS concentrations were generally higher than fine root levels across all species and seasons, with the exception of SS in mixed stands of both species during the rainy season (**Figures 2a, b, d, e**). Although CAT activity also varied among organs (*P* < 0.01, **Table 2**), its pattern depended on season, species, and stand type (**Figures 1c, f, i**). MDA concentration showed few organ‐level differences (*P* > 0.05), except for monospecific *L. racemosa* during the rainy season, where coarse roots exceeded fine roots and leaves, and mixed *A. marina* during the dry season, where leaves surpassed coarse roots (**Figures 2c, f, i**).

### 3.3 Effects of species, forest type, and stand type on physiological, morphological and anatomical traits

Significant dry−season variations in soil nutrients were observed across forest types (**Table 1**). Soil N was highest under *A. marina*, intermediate in the mixed stand, and lowest under *L. racemosa*. Conversely, the soil C:N ratio was significantly higher in the *L. racemosa* stand than in the *A. marina* and mixed stands, which did not differ from each other.

SOD, POD, and CAT activities were significantly affected by the forest type, defined by the combination of species and stand types (i.e., *L. racemosa* monoculture, *L. racemosa* mixed stand, *A. marina* monoculture, *A. marina* mixed stand, *P* < 0.01, **Table 2**). During the dry season, *L. racemosa* exhibited significantly higher SOD activity than *A. marina* in leaves and coarse roots across all stands, and in fine roots except in monospecific stands (*P* < 0.05, **Figures 1a, d, g**). Conversely, its POD activity in the dry season was lower than *A. marina* in leaves, fine roots, and coarse roots, except that coarse root levels were similar to *A. marina* in mixed stands (**Figures 1b, e, h**). In the wet season, *A. marina* fine and coarse roots had significantly lower CAT than *L. racemosa*. In the dry season, *A. marina* leaf CAT (11.0−11.5 U/g) significantly exceeded *L. racemosa* (3.8−4.7 U/g, **Figure 1c**). Differences in SS and PRO between species depended on season, organ, and stand type (**Figure 2**). MDA was also species‐dependent (**Table 2**), apart from a few organs and seasons, *A. marina* generally had significantly lower MDA than *L. racemosa* (**Figures 2c, f, i**).

Beyond these inter-specific variations, significant stand-type effects were also observed across morphological, anatomical, and physiological dimensions. During the dry season, *L. racemosa* leaf area was significantly higher in mixed than in monospecific stands, avoiding the sharp seasonal decline observed in the latter; conversely, *A. marina* leaf area remained stable (**Figure 3**). Anatomically, dry-season mixed stands significantly increased both conduit number and diameter in *L. racemosa* coarse roots, whereas only conduit number was elevated in *A. marina*, relative to monospecific stands (**Figures 4c, d**). Furthermore, *L. racemosa* maintained larger fine-root conduits in mixed stands year-round (**Figure 4b**), while wet-season *A. marina* mixed stands featured fewer but wider coarse-root conduits compared to monospecific stands (**Figures 4c, d**). Corresponding to these structural shifts, dry-season *L. racemosa* in mixed stands exhibited higher leaf PRO and fine-root CAT relative to monospecific stands (**Figures 1f**, **2b**), while other markers (e.g., coarse-root SOD and SS, **Figures 1g**, **2g**) remained stable or decreased. Finally, wet-season *A. marina* coarse-root SS was significantly higher in mixed than in monospecific stands (**Figure 2g**).

Within the supplementary LMM framework (**Supplementary Tables 2**, **3**), tree species identity had the broadest effect, significantly influencing most physiological traits (SOD, POD, CAT, PRO, and MDA) and nearly all morphological and anatomical traits (except coarse−root conduit number). In contrast, stand type (monospecific vs. mixed) significantly affected leaf area, fine- and coarse-root conduit diameter, and coarse-root conduit number. Significant species × stand−type interactions were detected for PRO, leaf area, fine− and coarse−root conduit diameter and number, and root N concentration. Furthermore, three-way interaction patterns differed essentially between trait categories. All physiological traits (SOD, POD, CAT, MDA, SS, PRO, N) showed non-significant season × species × stand type interactions (*P* > 0.05), whereas morphological and anatomical traits, including leaf area, and coarse root conduit number and diameter, exhibited significant three-way interactions (**Supplementary Tables 2**, **3**).

### 3.4 Correlations among physiological, morphological and anatomical traits

**Figure 5:**
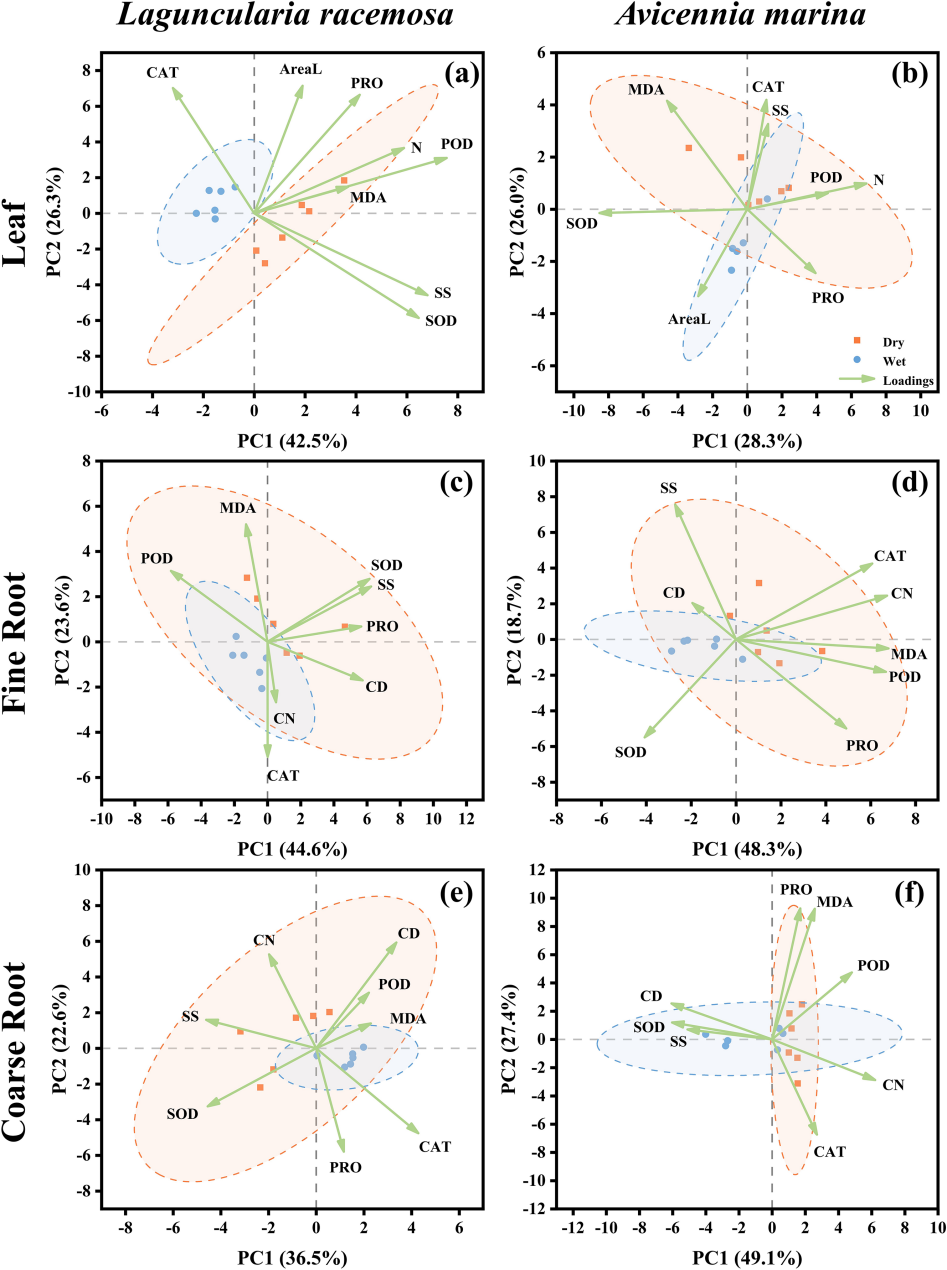
Principal component analysis visualizing the relationship between physiological traits (SOD, POD, CAT, SS, PRO, MDA) and organ-specific morphological variables in *Laguncularia racemosa* and *Avicennia marina*. Data from monospecific and mixed stands (*n* = 6 of each) were pooled for analysis. The analysis incorporated leaf area (AreaL) and tissue nitrogen concentration (N) for leaves **(a, b)**, whereas conduit number (CN) and diameter (CD) were used for fine roots **(c, d)** and coarse roots **(e, f)**. Plot-level independent replicates are represented by orange squares for the dry season and blue circles for the wet season. Shaded ellipses depict the 95% confidence intervals for each seasonal grouping. Vector arrows indicate the direction and strength of variable loadings on the principal components.

Patterns of correlation among MDA, antioxidant enzymes, and osmolytes differed by season and species (**Table 4**, **Figure 5**). A significant negative correlation between MDA and antioxidant defense was observed only in *A. marina* roots during the wet season (MDA vs. SOD: *r* = -0.917 and -0.831 for fine and coarse roots, respectively). In contrast, this negative regulation was absent in the dry season. Instead, the dry season was characterized by strong positive correlations between enzymatic activities and osmolyte accumulation. Specifically, PRO concentration correlated positively with SOD activity in *L. racemosa* fine roots (*r* = 0.933) and with POD activity in *A. marina* coarse roots (*r* = 0.825). Furthermore, in leaves, CAT activity in *A. marina* showed a strong positive linkage with SS concentration (*r* = 0.885), while POD activity in *L. racemosa* correlated positively with PRO concentration (*r* = 0.952). Specifically, these positive enzyme–osmolyte associations were stronger in leaves than in roots during the dry season (**Table 4**).

**Table 4:** Pearson correlation coefficients (*r*) for leaves, fine roots, and coarse roots of *Laguncularia racemosa* and *Avicennia marina* across seasons (*n* = 6).

Species	Season	Leaf	Fine root	Coarse root
*Laguncularia racemosa*	Wet season	POD&CAT (0.981 **)	POD&SOD (-0.825 *)	POD&SS (-0.899 *)
	Dry season	POD&PRO (0.952 **)	POD&CAT (-0.904 *) SOD&PRO (0.933 **) SS&PRO (0.815 *)	CAT&PRO (0.816 *)
*Avicennia marina*	Wet season	SOD&SS (-0.921 **) POD&SS (0.854 *)	POD&SS (-0.961**) SS&MDA (-0.829 *) SOD&MDA (-0.917 *) POD&MDA (0.816 *)	SOD&SS (0.875 *) SOD&MDA (-0.831 *)
	Dry season	SOD&PRO (-0.973 **) CAT&SS (0.885 *) SOD&MDA (0.843*)	SOD&POD (0.992 **) PRO&MDA (0.818 *)	POD&PRO (0.825 *) PRO&MDA (0.898 *)

Only significant relationships (*P* < 0.05) between indicators are shown; non-significant correlations are omitted. Abbreviations defined in **Figures 1** and **2** (*n* = 6). * and ** denote significant relationships at *P* < 0.05 and *P* < 0.01, respectively.

The PCA results, characterized by the distinct separation of 95% confidence ellipses between seasons across all tissues, reflect a multivariate pattern consistent with environmental seasonality rather than background ontogeny (**Figure 5**). In leaf tissues, loading vectors displayed species-specific geometry where *L. racemosa* leaf N, AreaL, POD, PRO, and MDA clustered in the dry-season quadrant, yet leaf N exhibited a tighter coupling with POD than with AreaL, which remained nearly orthogonal to SS and SOD (**Figure 5a**). Conversely, *A. marina* leaf N and POD were nearly co-linear and acute to CAT, SS, and PRO, while AreaL and SOD were oriented almost perpendicular to or opposite of this N-defense cluster (**Figure 5b**). The root systems of *L. racemosa* exhibited distinct trait−alignment patterns across root classes. In fine roots, CD oriented toward the dry-season quadrant while CN aligned with the PC2 axis (**Figure 5c**). In coarse roots, CD aligned with POD and MDA while opposing SOD, whereas CN was positioned nearly orthogonally to both MDA and SOD (**Figure 5e**). At the whole-root level, root N, CN, CD, SOD, and osmolytes collectively clustered within the dry-season quadrant, whereas the positive correlation between root N and CD was weaker than its association with CN and osmolytes (**Supplementary Figure S2a**). *A. marina* exhibited consistent structural-metabolic decoupling across all root scales, with CN clustering with dry-season stress markers (CAT, POD) and CD opposingly associated with the wet season (**Figures 5d, f**; **Supplementary Figure S2b**).

## 4 Discussion

### 4.1 Seasonal metabolic defense in mangroves: preventive strategy and nitrogen availability

Understanding how both native and invasive mangrove species respond to seasonal variations is central to predicting ecosystem resilience under climate change. In this study, the dry season strongly activate both antioxidant defenses and osmotic adjustment systems in both species. Specifically, the dry season induced significant increases in SOD activity and SS concentrations in *L. racemosa* leaves and roots, while elevating leave and root CAT, along with root POD levels, in *A. marina*. Furthermore, these enzymatic activities correlated with osmolyte accumulation: fine root SOD in *L. racemosa* with PRO, and leaf CAT in *A. marina* with SS, while coarse-root POD correlated with PRO. Similar seasonal upregulation of enzyme activity has been reported in tropical forests in Brazil (Boanares et al., 2020).

The significant activation of antioxidant defenses during the dry season, coupled with stable MDA levels, underscores a well-maintained redox equilibrium in both species. Crucially, as soil stress indicators (*e.g*., water content) relatively remained stable, this equilibrium was achieved without an increase in seasonal environmental pressure. The upregulation of enzymes in the absence of intensified stress, together with the general lack of negative enzyme–MDA correlations, is therefore consistent with a “preventive defense” model. This suggests that plants proactively strengthen their antioxidant systems to maintain ROS homeostasis, thereby suppressing lipid peroxidation before it initiates (Miller et al., 2010; Morales et al., 2016). While alternative mechanisms, such as seasonal differences in MDA turnover or increased enzyme activity compensating for higher ROS production, could also result in no net change in MDA (Zhao et al., 2017; Mittler et al., 2022), these typically involve dynamic damage–repair cycles that are unlikely to be captured by our single-time-point assessment. Consequently, preventive defense represents a plausible explanation for our observed data. Future multi-temporal studies focusing on MDA degradation markers are required to further validate these complex metabolic dynamics.

While our field-based approach captures authentic ecological responses, it introduces two key methodological constraints. First, seasonal fluctuations in tissue hydration may bias fresh-weight (FW)-normalized metrics. Despite a limited validation sample size (*n* = 2 composites per class), tissue water content remained stable across seasons, consistent with concurrent soil moisture stability. This stability aligns with prior reports of seasonal leaf water constancy in *L. racemosa* and the congeneric *Avicennia germinans* (Sobrado and Ewe, 2006), providing a comparative baseline. To evaluate the potential impact of hydration shifts, a sensitivity analysis recalculating enzymatic activities into dry-weight (DW) equivalents (**Supplementary Figure S1**) was conducted as a robustness check. Because this recalculation relies on the same limited hydration coefficients (*n* = 2) employed in the pooled confidence intervals, and thus shares the inherent circularity acknowledged in the Methods section, it cannot serve as an independent empirical validation of tissue water stability. Rather, it functions as a mathematical consistency check: the persistence of all significant seasonal differences under DW normalization, alongside the detection of three additional enzymatic upregulations, indicates that the FW-based estimates are conservative rather than inflated. This consistency demonstrates that the observed seasonal patterns are robust to alternative normalization, despite the inability to independently validate tissue water stability with the current dataset. Second, seasonal metabolic shifts might be confounded by concurrent ontogenetic development. However, expected cumulative growth, such as consistent expansion of leaf area and root conduits, was largely absent over the six-month study, except in mixed *L. racemosa*. This was mirrored in our PCA, where leaf area vectors separated from the seasonal physiological cluster, and root conduit traits showed divergent alignments, indicating that ontogeny alone is unlikely to fully explain the synchronized metabolic shifts. To statistically evaluate these visual trends, LMMs (**Supplementary Tables 2**, **3**) were used to explore a potential divergence in the interaction architecture between morphological and physiological traits. Specifically, morphological and anatomical traits, such as leaf area and coarse root conduit traits, exhibited highly significant three-way interactions (season × species × stand type), indicating that ontogenetic trajectories are strictly context-dependent. In contrast, none of the physiological markers (*e.g*., SOD, MDA, PRO) exhibited such higher-order interactions, despite being analyzed within the identical model structure. This was further supported by the fact that key ontogenetic markers, including leaf area and fine root conduit diameter, showed no significant seasonal main effects. Collectively, these patterns are consistent with the possibility that metabolic adjustments represent primary adaptations to seasonal cues rather than developmental artifacts. Nevertheless, as the absence of significant interactions and main effects may reflect limited statistical power (*n* = 3), the results point toward this interpretation but do not firmly establish seasonal control.

Given that the observed shifts align with seasonal transitions, exploring potential environmental drivers may provide further insight into the underlying mechanisms. The stability of soil moisture and salinity suggests that the seasonal physiological shifts are independent of water or salt stress, pointing instead to soil N availability. Specifically, increased soil N concentration and lowered C:N ratios during the dry season are interpreted as evidence for greater mineralization than immobilization and less leaching (Elrys et al., 2021), which could, in principle, facilitate the allocation of surplus N to the production of N-intensive antioxidants and osmolytes (Iqbal et al., 2020). However, the mechanistic link between soil N availability and defensive up-regulation is strictly conceptual rather than causal due to several missing key links. First, without direct soil process measurements (*e.g*., ^15^N methods), we cannot definitively separate mineralization from immobilization or quantify actual N flux. Second, the fact that tissue N shows little seasonal change in most cases (except *A. marina* monocultures) directly weakens the argument that greater N uptake drives defensive investment. Furthermore, most evidence supporting N-induced antioxidant responses originates from controlled fertilization studies (Chen and Ye, 2014; Farzana et al., 2019), which often fail to replicate the naturally varying N levels of complex field environments. Consequently, while the observed correspondence between tissue N and defensive systems (relative to growth traits) provides a suggestive signal of preferential N allocation toward defense, the above constraints mean that the proposed N-surplus mechanism cannot be confirmed and should be treated strictly as a working hypothesis that requires further testing.

### 4.2 Organ-specific differences in adaptive strategies between *A. marina* and *L. racemosa*

In mangrove ecosystems, leaves and roots serve distinct functions and thus employ different physiological responses under stress. However, comparative analyses of organ-level responses in invasive versus native species remain limited. Our analysis showed that, across both species, leaves generally exhibited higher antioxidant activities than roots (*e.g*., higher SOD in *L. racemosa* during the dry season and higher POD in *A. marina* across seasons), and osmolyte (PRO and SS) levels were also typically higher in leaves than in fine roots during the dry season, while MDA levels remained similar between organs. One possible explanation for this pattern is that leaves, as photosynthetic tissues, are more exposed to light-induced ROS production in chloroplasts, which increases the risk of PSII photodamage under stress conditions (Cruz de Carvalho, 2008; Miller et al., 2010; Xiong et al., 2021). This heightened oxidative stress in leaves may drive the observed increase in antioxidant and osmolyte levels comparable to those in roots. From a whole-plant perspective, this spatial partitioning suggests that defensive investments are preferentially allocated to highly exposed tissues, providing a shared mechanism for both species to manage local oxidative risks. However, as we did not directly measure chloroplast ROS, PSII function, or photosynthetic performance, these functional interpretations remain to be verified in future studies.

Dry-season conditions induced coordinated morphological and anatomical adjustments that complemented biochemical defenses. Stable or reduced leaf area during the dry season (except in mixed *L. racemosa*), while potentially essential for preventing hydraulic failure, may limit photosynthetic capacity (Zhao and Running, 2010; López et al., 2021). Concurrently, higher soil N concentration during the dry season is consistent with enhanced root metabolic activities, potentially mediated by the synthesis of N-intensive enzymes and osmolytes (Iqbal et al., 2020), such as PRO and amino acids. At the anatomical level, roots of both species consistently increased conduit number rather than conduit diameter. Given that wider conduits, while efficient for water transport, are highly susceptible to drought-induced embolism compared to thinner conduits (Choat et al., 2012; Anderegg et al., 2016), this adjustment maintained hydraulic conductivity by expanding the total conductive area while minimizing hydraulic vulnerability. Therefore, across the root-leaf continuum, these structural adjustments closely complement internal biochemical defenses to manage the fundamental trade-offs between acquisition and safety. Addressing our initial inquiry, this broad organ-level analysis reveals that conservative structural adjustments that complement internal defenses serve as a fundamental physiological baseline for both the invasive and native species to buffer seasonal stress.

### 4.3 Species- and stand-type specific physiological adjustments to seasonal dynamics

*L. racemosa* and *A. marina* exhibited species-specific differences in stress-response traits, with *L. racemosa* generally maintaining higher MDA concentration and SOD activity (during the dry season) but lower POD activity compared to *A. marina*. Yet, intrinsic variations in ROS generation rates, and water-use efficiency between species limit the value of absolute comparisons (Boanares et al., 2020; Zhou et al., 2021). Instead, examining the magnitude of seasonal changes within each species better illuminates their responsive capacity by revealing divergent response strategies.

These distinct physiological adjustments were most pronounced in leaves, which are directly exposed to atmospheric drivers. Generally, mild oxidative stress activates SOD and POD, whereas severe stress tends to trigger CAT dominance (Cruz de Carvalho, 2008; Wang et al., 2022). While short−term experiments with single stressors (*e.g*., chilling or heavy metals) have consistently reported increased leaf SOD activity in both species (Zhou et al., 2021; Lang et al., 2022; Wang et al., 2022), our results from the natural dry season, characterized by prolonged and combined stresses, revealed divergent response patterns. *L. racemosa* exhibited a sustained increase in leaf SOD activity across stand types, whereas under the same conditions, *A. marina* showed marked upregulation of leaf CAT activity. This aligns with prior studies indicating that prolonged or combined stress in *A. marina* elevates the role of CAT in leaf ROS detoxification (Zhou et al., 2021; Wang et al., 2022). These patterns hold under examined conditions but require broader environmental testing. Nevertheless, these findings underscore a critical distinction: physiological models derived from controlled, acute stress scenarios may fail to predict the divergent enzymatic regulation pathways employed by these species under the complex, chronic conditions of natural environments.

The divergent functional responses also manifested in roots, where significant interactions between species and stand type drove species-specific shifts in root conduit traits and N concentration, alongside coordinated adjustments in leaf area and PRO. A key finding was that *L. racemosa* significantly expanded its coarse root conduit diameter in dry-season mixed stands relative to its monoculture, whereas this response was not observed in *A. marina*. This disparity is mechanistically rooted in species-specific plasticity in response to stand-type-mediated stress gradients. Specifically, during the dry season, soil salinity in mixed stands was significantly lower than in *L. racemosa* monocultures, thereby triggering its acquisitive strategy (Wei et al., 2022). Mechanistically, this environmental release enables *L. racemosa* to leverage xylem plasticity to relax the typical constraint of conduit narrowing required for cavitation safety (Reef and Lovelock, 2015), facilitating the development of wider conduits in these lower-salinity environments to maximize hydraulic efficiency. Notably, our results show this coarse root expansion synchronized with increased leaf area and N concentration, indicating that enhanced transport capacity is coordinated to accommodate the high transpirational demands of a high-stomatal-density canopy (Drake et al., 2013; Bai et al., 2023) and to efficiently capitalize on higher soil N availability relative to its monoculture, thereby facilitating competitive growth (Cardona-Olarte et al., 2006). Importantly, this stand-specific shift toward high-risk acquisitive traits does not conflict with the conservative baseline identified at the broader organ level (Section 4.2); rather, it highlights the invader’s distinct plasticity to selectively deviate from this physiological foundation when localized environmental constraints are relaxed. Conversely, coarse root diameter remained stable across *A. marina* stand types in the dry season, mirroring the absence of significant salinity fluctuation observed in its mixed stands. Functionally, increasing conduit number while maintaining diameter in coarse roots reflects a conservative hydraulic architecture, which prioritizes hydraulic safety to effectively minimize metabolic costs (Choat et al., 2012; Anderegg et al., 2016). The resulting limited transport efficiency of this conservative strategy, compounded by the lower soil N availability in mixed stands compared to *A. marina* monocultures, directly explains the significant decline in leaf N concentration found in our study.

The significant effect of stand type highlights a complex interplay between native-mediated facilitation and competitive pressure. In mixed stands, the presence of native *A. marina* appears to be associated with edaphic improvement relative to *L. racemosa* monocultures, specifically via reduced salinity and enhanced N availability (Santonja et al., 2017; Bajahmoum and Almaghamsi, 2025), thereby mitigating abiotic stress exposure for *L. racemosa*. However, this environmental release imposes a critical constraint: to efficiently capitalize on these enriched resources while concurrently co-existing with the stress-tolerant native, *L. racemosa* effectively exhibits a shift to a resource-intensive, high-risk functional regime. This physiological shift is characterized by the formation of wide, embolism-prone conduits and substantial PRO accumulation, serving as metabolically expensive compensatory traits required to sustain competitive superiority. Collectively, these findings align with biotic resistance theory, suggesting that native community structure influences invasive species physiology beyond simple presence or absence effects (Funk and Vitousek, 2007; Beaury et al., 2020) by imposing costly physiological trade-offs.

## 5 Conclusion

Our field-based study proposes a preventive defense model as a plausible physiological framework to explain the observed seasonal patterns, wherein both species significantly upregulate antioxidant enzymes and osmolytes during the dry season despite relatively stable soil stress indicators (*e.g*., water content). In this hypothesized model, the observed enzymatic shifts are interpreted as a proactive mechanism to maintain redox homeostasis. However, as sampling only once per season limits the ability to distinguish seasonal conditions from dynamics, this biologically plausible preventive defense model cannot be clearly differentiated from a single snapshot of ongoing damage-repair processes. Consequently, our interpretation remains a hypothesis requiring future validation through repeated temporal measurements of metabolic degradation markers. Multivariate associations derived from PCA further reveal a metabolic prioritization of defense over plant growth across organ-specific trait syndromes. However, these results require confirmation through direct correlation or regression analyses; the current patterns should be interpreted as exploratory rather than confirmatory. This whole-plant physiological regulation is spatially coordinated: leaves maximize antioxidative capacity to mitigate atmospheric radiative stress, while roots modulate hydraulic architecture, establishing a functional divergence between organ-specific biochemical defense and hydraulic transport roles.

Specifically, interspecific interactions shape divergent hydraulic strategies. *A. marina* maintains a conservative strategy across stand types, prioritizing hydraulic safety by increasing root conduit number. In contrast, *L. racemosa* exhibits a shift toward hydraulic efficiency specifically within mixed stands relative to monocultures. In these mixed stands, facilitative effects for *L. racemosa*, likely manifesting through reduced soil salinity, coincide with an acquisitive strategy characterized by the expansion of coarse root conduit diameter. This anatomical expansion theoretically enhances hydraulic capacity to support the transport of abundant soil N toward the canopy. However, this shift imposes a critical constraint: the invasion success of *L. racemosa* is contingent upon sustaining this high-risk (*i.e*., embolism-prone) and metabolically expensive physiological regime. Together, this contrast provides mechanistic support for biotic resistance, suggesting that native community structure constrains invasion by forcing invaders into risky physiological compromises to sustain competitive dominance.

## Data Availability

The raw data supporting the conclusions of this article will be made available by the authors, without undue reservation.
