# Supplementary material for: Distinct physiological and functional responses in leaves and roots of co-occurring mangroves: contrasting invasive Laguncularia racemosa and native Avicennia marina under seasonal nutrient fluctuations
Source: Front Plant Sci. 2026 Mar 30;17:1772780. doi: 10.3389/fpls.2026.1772780 (PMC13070957; doi:10.3389/fpls.2026.1772780)
Supplement: Supplementary file 1 [file DataSheet1.docx]

| **Season** | **Forest type** | **Leaf** | **Root** |
| --- | --- | --- | --- |
| Wet season | LR-P | 5.05 ± 0.38 Xz | 1.41 ± 0.06 Yy |
|  | LR-M | 6.97 ± 0.26 Xy | 1.15 ± 0.12 Yy |
|  | AM-P  AM-M | 15.16± 0.37 Xx*  15.56± 0.57 Xx | 4.39 ± 0.21 Yx  4.14± 0.23 Yx |
|  |  |  |  |
| Dry season | LR-P | 6.42 ± 0.58 Ad | 1.63 ± 0.09 Bb |
|  | LR-M | 8.48 ± 0.71 Ac | 2.55 ± 0.36 Bb |
|  | AM-P  AM-M | 18.83± 1.8 Aa*  15.37± 0.71 Ab | 5.7± 0.85 Ba  5.09± 0.79 Ba |

**Table S1** Seasonal variation in nitrogen concentration of leaves and roots in monospecific and mixed stands of *Laguncularia racemosa* and *Avicennia marina* during dry and wet seasons.

LR-P, *Laguncularia racemosa* *monospecific stand*; LR-M, *Laguncularia racemosa mixed stand*; AM-P, *Avicennia marina monospecific stand*; AM-M, *Avicennia marina mixed stand.* Different lowercase letters denote significant differences among the four stand-species combinations within the same organ and season, x, y, z for wet season, a, b, c, d for dry season; uppercase letters denote differences among organs within the same stand-species combination and season, X, Y for wet season, A, B for dry season. Asterisks (*) indicate seasonal differences within the same stand-species combination and organ (*P* < 0.05).


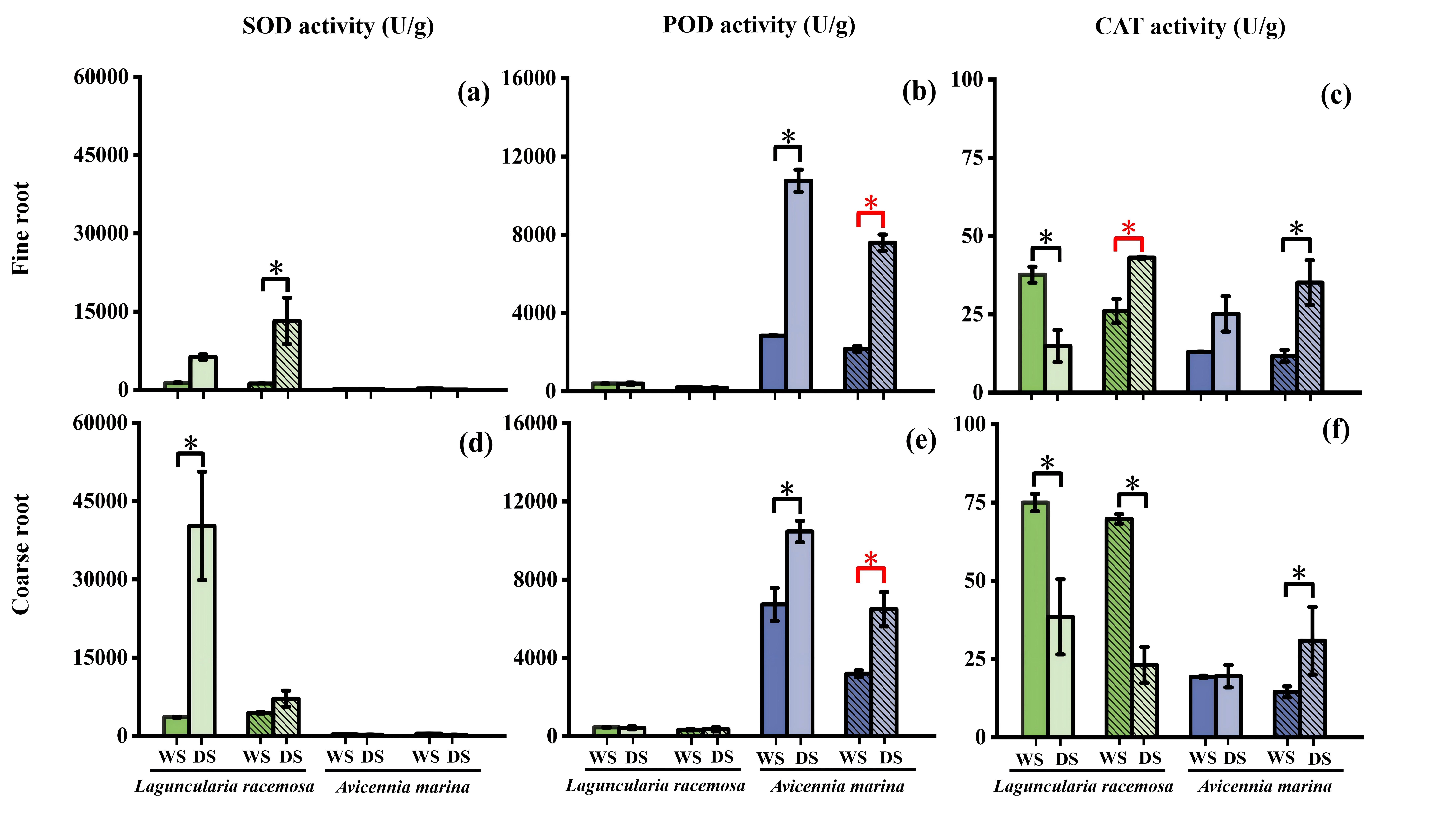


**Fig. S1** Sensitivity analysis recalculating superoxide dismutase (SOD), peroxidase (POD), and catalase (CAT) activities from fresh-weight (FW) to dry-weight (DW) equivalents. Data represent fine (a-c) and coarse (d-f) roots of *Laguncularia racemosa* and *Avicennia marina* within monospecific and mixed stands during the wet and dry seasons (*n* = 3). Red markers indicate additional significant seasonal differences revealed by DW normalization compared to initial FW analyses (Fig. 1).


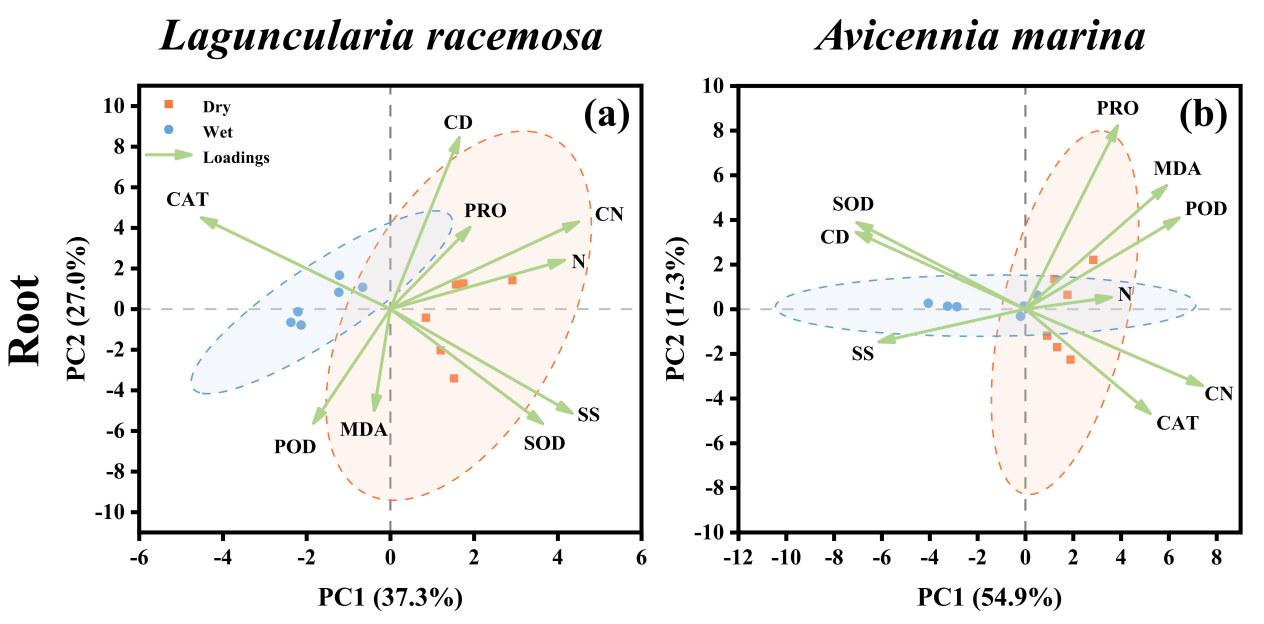


**Fig. S2** Principal component analysis visualizing the relationships among physiological traits (SOD, POD, CAT, SS, PRO, MDA) and anatomical and histochemical variables in *Laguncularia racemosa* (a) and *Avicennia marina* (b) roots. Data from monospecific and mixed stands (*n* = 6 of each) were pooled for analysis. The anatomical and histochemical variables included root conduit number (CN), conduit diameter (CD), and tissue nitrogen concentration (N). Plot-level independent replicates are represented by orange squares for the dry season and blue circles for the wet season. Shaded ellipses depict the 95% confidence intervals for each seasonal grouping. Vector arrows indicate the direction and strength of variable loadings on the principal components.

**Table S2** Linear mixed model results for the main and interactive effects of season, tree species, and stand type (monospecific vs. mixed) on SOD, POD, CAT activities, as well as SS, PRO, and MDA concentrations in *Laguncularia racemosa* and *Avicennia marina*.

| **Variables** | **Values** | **Season** | **Species** | **Stand Type** | **Season × Species** | **Season × Stand Type** | **Species × Stand Type** | **Season × Species × Stand Type** |
| --- | --- | --- | --- | --- | --- | --- | --- | --- |
| SOD | *F* value | 20.77 | 33.61 | 0.93 | 21.48 | 1.19 | 1.25 | 1.22 |
|  | *P* value | ***P*<0.01** | ***P*<0.01** | 0.34 | ***P*<0.01** | 0.28 | 0.27 | 0.27 |
|  |  |  |  |  |  |  |  |  |
| POD | *F* value | 0.58 | 42.70 | 1.76 | 0.43 | 0.01 | 1.97 | 0.02 |
|  | *P* value | 0.45 | ***P*<0.01** | 0.19 | 0.51 | 0.93 | 0.17 | 0.90 |
|  |  |  |  |  |  |  |  |  |
| CAT | *F* value | 2.77 | 4.46 | 3.17 | 35.59 | 0.00 | 0.83 | 0.88 |
|  | *P* value | 0.10 | **0.04** | 0.08 | ***P*<0.01** | 0.99 | 0.37 | 0.35 |
|  |  |  |  |  |  |  |  |  |
| MDA | *F* value | 4.30 | 59.56 | 3.10 | 1.33 | 1.33 | 1.92 | 0.48 |
|  | *P* value | **0.04** | ***P*<0.01** | 0.11 | 0.25 | 0.25 | 0.18 | 0.49 |
|  |  |  |  |  |  |  |  |  |
| PRO | *F* value | 0.31 | 5.17 | 0.77 | 0.00 | 0.28 | 4.83 | 2.59 |
|  | *P* value | 0.58 | **0.03** | 0.38 | 0.99 | 0.60 | **0.03** | 0.11 |
|  |  |  |  |  |  |  |  |  |
| SS | *F* value | 8.79 | 1.41 | 0.00 | 10.64 | 0.79 | 0.79 | 0.00 |
|  | *P* value | ***P*<0.01** | 0.24 | 1.00 | ***P*<0.01** | 0.38 | 0.38 | 1.00 |

**Table S3** Linear mixed model results for the main and interactive effects of season, tree species, and stand type (monospecific vs. mixed) on leaf and root morphological, anatomical, and biochemical traits in *Laguncularia racemosa* and *Avicennia marina*.

| **Organs** | **Variables** | **Values** | **Season** | **Species** | **Stand Type** | **Season × Species** | **Season × Stand Type** | **Species × Stand Type** | **Season × Species × Stand Type** |
| --- | --- | --- | --- | --- | --- | --- | --- | --- | --- |
| Leaf | Leaf Area | *F* value | 1.84 | 29.33 | 10.15 | 0.20 | 11.12 | 9.10 | 12.85 |
|  |  | *P* value | 0.19 | ***P*<0.01** | **0.01** | 0.66 | ***P*<0.01** | **0.01** | ***P*<0.01** |
|  | Leaf total nitrogen | *F* value | 9.44 | 98.98 | 0.02 | 0.26 | 0.43 | 1.43 | 1.46 |
|  |  | *P* value | **0.01** | ***P*<0.01** | 0.88 | 0.62 | 0.52 | 0.25 | 0.25 |
| Root | *Pooled root* |  |  |  |  |  |  |  |  |
|  | Total nitrogen | *F* value | 5.61 | 358.24 | 0.12 | 0.09 | 1.93 | 12.33 | 3.99 |
|  |  | *P* value | **0.04** | ***P*<0.01** | 0.74 | 0.77 | 0.20 | ***P*<0.01** | 0.07 |
|  | *Fine root* |  |  |  |  |  |  |  |  |
|  | Conduit diameter | *F* value | 3.01 | 129.52 | 29.69 | 5.52 | 0.10 | 36.78 | 2.34 |
|  |  | *P* value | 0.10 | ***P*<0.01** | ***P*<0.01** | **0.03** | 0.75 | ***P*<0.01** | 0.15 |
|  | Conduit number | *F* value | 16.98 | 146.95 | 0.07 | 71.29 | 0.33 | 5.70 | 1.30 |
|  |  | *P* value | ***P*<0.01** | ***P*<0.01** | 0.79 | ***P*<0.01** | 0.58 | **0.03** | 0.27 |
|  | *Coarse root* |  |  |  |  |  |  |  |  |
|  | Conduit diameter | *F* value | 15.93 | 78.81 | 67.67 | 2.39 | 2.93 | 28.03 | 81.73 |
|  |  | *P* value | ***P*<0.01** | ***P*<0.01** | ***P*<0.01** | 0.14 | 0.11 | ***P*<0.01** | ***P*<0.01** |
|  | Conduit number | *F* value | 438.88 | 0.66 | 97.76 | 0.10 | 24.17 | 206.20 | 100.81 |
|  |  | *P* value | ***P*<0.01** | 0.43 | ***P*<0.01** | 0.76 | ***P*<0.01** | ***P*<0.01** | ***P*<0.01** |
